# Milk fat globule EGF factor 8 restores mitochondrial function via integrin‐medicated activation of the FAK‐STAT3 signaling pathway in acute pancreatitis

**DOI:** 10.1002/ctm2.295

**Published:** 2021-01-24

**Authors:** Yifan Ren, Wuming Liu, Lin zhang, Jia Zhang, Jianbin Bi, Tao Wang, Mengzhou Wang, Zhaoqing Du, Yawen Wang, Lin zhang, Zheng Wu, Yi Lv, Lingzhong Meng, Rongqian Wu

**Affiliations:** ^1^ National Local Joint Engineering Research Center for Precision Surgery & Regenerative Medicine Shaanxi Provincial Center for Regenerative Medicine and Surgical Engineering First Affiliated Hospital of Xi'an Jiaotong University. Xi'an Shaanxi Province China; ^2^ Department of Hepatobiliary Surgery First Affiliated Hospital of Xi'an Jiaotong University. Xi'an Shaanxi Province China; ^3^ Biobank First Affiliated Hospital of Xi'an Jiaotong University. Xi'an Shaanxi Province China; ^4^ Department of Laboratory Medicine First Affiliated Hospital of Xi'an Jiaotong University Xi'an Shaanxi Province China; ^5^ Department of Anesthesiology Yale University School of Medicine New Haven Connecticut USA

**Keywords:** acute pancreatitis, FAK‐STAT3, MFG‐E8, mitochondrial function, outcome

## Abstract

**Abstract:**

Acute pancreatitis (AP) remains a significant clinical challenge. Mitochondrial dysfunction contributes significantly to the pathogenesis of AP. Milk fat globule EGF factor 8 (MFG‐E8) is an opsonizing protein, which has many biological functions via binding to αvβ3/5 integrins. Ligand‐dependent integrin‐FAK activation of STAT3 was reported to be of great importance for maintaining a normal mitochondrial function. However, MFG‐E8's role in AP has not been evaluated.

**Methods:**

Blood samples were acquired from 69 healthy controls and 134 AP patients. Serum MFG‐E8 levels were measured by ELISA. The relationship between serum concentrations of MFG‐E8 and disease severity were analyzed. The role of MFG‐E8 was evaluated in experimental models of AP.

**Results:**

Serum concentrations of MFG‐E8 were lower in AP patients than healthy controls. And serum MFG‐E8 concentrations were negatively correlated with disease severity in AP patients. In mice, MFG‐E8 administration decreased L‐arginine‐induced pancreatic injury and mortality. MFG‐E8's protective effects in experimental AP were associated with improvement in mitochondrial function and reduction in oxidative stress. MFG‐E8 knockout mice suffered more severe pancreatic injury and greater mitochondrial damage after l‐arginine administration. Mechanistically, MFG‐E8 activated the FAK‐STAT3 pathway in AP mice. Cilengitide, a specific αvβ3/5 integrin inhibitor, abolished MFG‐E8's beneficial effects in AP. PF00562271, a specific FAK inhibitor, blocked MFG‐E8‐induced STAT3 phosphorylation. APTSTAT3‐9R, a specific STAT3 antagonist, also eliminated MFG‐E8's beneficial effects under such a condition.

**Conclusions:**

MFG‐E8 acts as an endogenous protective mediator in the pathogenesis of AP. MFG‐E8 administration protects against AP possibly by restoring mitochondrial function via activation of the integrin‐FAK‐STAT3 signaling pathway. Targeting the action of MFG‐E8 may present a potential therapeutic option for AP.

## Graphical abstract

GRAPHICAL HEADLIGHTS

Serum MFG‐E8 levels were negatively correlated with disease severity in patients with acute pancreatitis;

MFG‐E8‐deficient mice suffered more severe pancreatic injury in experimental acute pancreatitis.

MFG‐E8 administration restores mitochondrial function in AP via activation of the integrin‐FAK‐STAT3 signaling pathway.

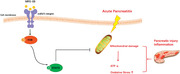

**ABBREVIATIONS**
AP: acute pancreatitis
APACHE II: Acute Physiology and Chronic Health Evaluation II
APTT: activated partial thromboplastin time
AST: aspartate aminotransferase
ATP: adenosine triphosphate
ATPB: ATPase beta subunits
AUC: area under the curve
BMI: body mass index
BUN: blood urea nitrogen
CI: confidence interval
Cr: creatinine
CRP: C‐reactive protein
DHE: dihydroethidium
FAK: focal adhesion kinase
FIB: fibrinogen
FRAP: ferric reducing antioxidant power
LDH: lactate dehydrogenase
LPS: lipopolysaccharide
MDA: manoldialdehyde
MFG‐E8: milk fat globule epidermal growth factor 8
Mfn‐2: mitofusin‐2
MODS: multiple organ dysfunction syndrome
ND3: NADH dehydrogenase subunit 3
OR: odds ratio
PGC‐1α: peroxisome proliferative activated receptor‐γ coactivator 1α
PLT: platelets
PT: prothrombin time
RIP3: receptor‐interacting protein kinase 3
ROC: receiver operating characteristic
ROS: reactive oxygen species
SOD: superoxide dismutase
SOFA: sequential organ failure assessment
STAT3: signal transduction and transcriptional activation factor 3
Tfam: mitochondrial transcription factor
TUNEL: TdT‐mediated dUTP Nick‐End Labeling
WBC: white blood cell

## 1 INTRODUCTION

Acute pancreatitis (AP) remains a significant clinical challenge.^1^ It is characterized by self‐destruction of the pancreatic tissue and rapid development of the inflammatory response.^2^, ^3^, ^4^ Severe AP, which accounts for about 20% of the cases, may lead to multiple organ dysfunction syndrome (MODS) and carries a high risk of mortality.^5^ Despite recent advances in intensive care, specific treatment for severe AP is still elusive, that is, current treatment is primarily palliative care.^6^ With growing incidences of AP worldwide, a novel effective treatment of people with severe AP is urgently needed.

Healthy mitochondria are vital for many biological functions of the pancreas.^7^ Extensive evidence from both clinical and experimental studies indicates that mitochondrial injury contributes significantly to the pathogenesis of AP.^3^, ^8^, ^9^ The abnormal mitochondrial function in the pancreas not only results in impairment of energy production but also leads to oxidative stress, which exaggerate pancreatic injury and inflammation.^10^ Disrupting the normal mitochondrial dynamics has been shown to trigger pancreatitis in various animal models.^11^ Maintaining the proper function of mitochondria, therefore, provides a strategic basis for many novel promising therapeutic approaches for AP.^12^, ^13^

Milk fat globule epidermal growth factor (EGF) factor 8 (MFG‐E8) is a secreted lipophilic glycoprotein.^14^, ^15^ It contains the RGD motif, which is known to interact with integrins. Several studies suggested that αvβ3 and αvβ5 integrins act as the MFG‐E8 receptor.^16^, ^17^, ^18^ Focal adhesion kinase (FAK) is a major integrin signaling mediator. Ligand‐dependent integrin‐FAK activation of STAT3 (signal transduction and transcriptional activation factor 3) was reported to be of great importance for maintaining a normal mitochondrial function.^19^ Aziz M. showed that MFG‐E8 activates the STAT3 signaling pathway in macrophages.^20^ However, MFG‐E8's role in AP has not been evaluated. We therefore hypothesized that MFG‐E8 is an endogenous protective mediator in AP and administration of exogenous MFG‐E8 attenuates mitochondrial damage in experimental AP via activation of the integrin‐FAK‐STAT3 signaling pathway.

We examined circulating concentrations of MFG‐E8 in patients with AP, analyzed the association between serum MFG‐E8 concentrations and disease severity, and investigated the effects and possible mechanism of MFG‐E8 treatment in experimental models of AP. MFG‐E8 knockout mice were utilized to verify the significance of MFG‐E8 in AP. The results would demonstrate the important role of MFG‐E8 in AP.

## 2 RESULTS

### 2.1 Serum MFG‐E8 concentrations are decreased in AP patients

**FIGURE 1:**
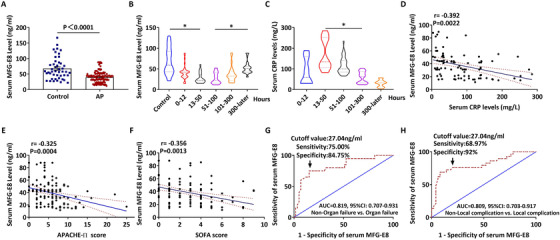
Serum MFG‐E8 levels were decreased in patients with acute pancreatitis. Blood samples from 134 acute pancreatitis patients (AP) and 69 healthy controls (Control) were collected, and serum MFG‐E8 levels were measured. A, Serum MFG‐E8 levels in AP patients and healthy controls; B, Serum MFG‐E8 levels in AP patients at different time of onset; C, Serum CRP levels in AP patients at different time of onset; D, Correlation analysis of serum MFG‐E8 levels and serum CRP levels; E, Correlation analysis of serum MFG‐E8 levels and APACHE II scores; F, Correlation analysis of serum MFG‐E8 levels and SOFA scores; G, ROC curve in AP patients with and without organ failure; H, ROC curve in AP patients with and without local complication. Error bars indicate the SEM; ∗ P < 0.05 versus control. APACHE II, Acute Physiology and Chronic Health Evaluation II; SOFA, Sequential Organ Failure Assessment; ROC, Receiver Operating Characteristic; CRP, C‐reactive protein

A total of 69 healthy controls and 134 AP patients were included in this study. Of these patients, 57 (42.5%) had biliary disease, 5 (3.7%) had alcohol misuse, 37 (27.6%) had hyperlipidemia, and 35 (26.1%) had other causes. Local complications occurred in 35 patients (26.1%), organ failure in 19 patients (14.2%), and in‐hospital mortality in 1 patient (0.7%) (Table S1). Compared with healthy controls, AP patients had significantly lower MFG‐E8 levels in the serum (Figure 1A, *P* < .0001). The mean MFG‐E8 serum level in healthy controls is 67.62 ng/mL. There is no sex‐specific difference of MFG‐E8 serum levels in healthy controls or AP patients. The time‐course study showed that serum MFG‐E8 levels decreased quickly at the early stage of AP and then started to rise at around 100 h after the onset of AP (Figure 1B, *P* < .05).

C‐reactive protein (CRP) is a commonly used circulating marker of inflammation. As shown in Figure 1C,D, serum MFG‐E8 concentrations were negatively correlated with serum CRP concentrations, suggesting an inverse relationship between serum MFG‐E8 levels and inflammatory severity. As shown in Figure 1E,F, serum MFG‐E8 concentrations were negatively correlated with APACHE II scores (*r* = ‐0.325, *P* < .01) and SOFA scores (*r* = ‐0.356, *P* < .01) in AP patients. Consistently, serum MFG‐E8 concentrations had an inversed correlation with the development of organ failure in AP patients (Figure 1G). The calculated cut‐off for serum MFG‐E8 levels to identify patients at risk of organ failure was 27.04 ng/mL, with a sensitivity of 75.0% and a specificity of 84.8%. The sensitivity and specificity to discriminate patients at risk of local complications were 68.9% and 92.0%, respectively (Figure 1H). As shown in Table 1, APACHE II scores and serum MFG‐E8 levels were independently associated with the development of organ failure in AP in the multivariate analysis.

**TABLE 1:** Univariable and multivariable analyses of risk factors for the development of organ failure in AP patients

			Univariate Analysis		Multivariable Analysis	
Variables	Non‐organ failure (*n* = 115)	Organ failure (*n* = 19)	*P*	OR (95% CI)	*P*	OR (95% CI)
**Sex (M/F)**	71/44	12/7	.438	1.46 (0.56‐3.81)		
Age (year)	48.52 ± 15.39	48.54 ± 12.54	.999	1.00 (0.96‐1.03)		
**BMI (kg/m^2^)**	21.94 ± 1.80	21.82 ± 1.97	.777	0.96 (0.74‐1.24)		
**Serum Glucose (mmol/L)**	7.48 ± 3.48	12.68 ± 5.88	<.001	1.28 (1.13‐1.45)	.385	1.15 (0.84‐1.56)
**HbA1c (%)**	5.22 ± 0.31	5.21 ± 0.36	.966	1.04 (0.24‐4.44)		
**Serum amylase (U/L)**	219 (103‐721)	210 (17‐1088)	.801	1.00 (0.99‐1.00)	.400	0.99 (0.98‐1.03)
**Serum lipase (U/L)**	855 (372‐2155)	1274 (229‐3133)	.791	1.05 (1.00‐1.00)	.243	1.01 (0.99‐1.02)
PLT (*10^9^/L)	197.1 ± 74.90	216.58 ± 95.37	.313	1.01 (0.99‐1.01)		
WBC (*10^9^/L)	10.01 ± 4.11	14.51 ± 8.19	.003	1.15 (1.05‐1.25)	.591	0.94 (0.72‐1.19)
**PT (S)**	14.89 ± 2.06	15.94 ± 2.29	.061	1.23 (0.99‐1.53)		
**FIB (g/L)**	4.58 ± 2.10	5.77 ± 1.95	.027	1.31 (1.03‐1.64)	.845	1.08 (0.50‐2.32)
**APTT (S)**	37.80 ± 5.67	36.47 ± 9.94	.424	0.97 (0.91‐1.04)		
**Serum K^+^ (mmol/L)**	3.95 ± 0.52	3.90 ± 0.61	.731	0.86 (0.36‐2.05)		
**Serum Na^+^ (mmol/L)**	138.94 ± 3.89	139.17 ± 4.63	.814	1.01 (0.91‐1.12)		
**Serum bilirubin (μmol/L)**	21.3 (16.1‐37.9)	38.3 (21.1‐72.9)	.008	1.03 (1.01‐1.05)	.237	0.98 (0.96‐1.00)
**Serum AST (mmol/L)**	30 (19‐45)	33.5 (18.2‐52.2)	.717	0.99 (0.99‐1.01)		
**Serum Cr (μmol/L)**	58 (46‐71)	62 (46‐91)	.118	1.01 (0.99‐1.13)		
**Serum BUN (mmol/L)**	5.88 ± 2.13	9.32 ± 9.10	.039	1.16 (1.00‐1.36)	.496	1.12 (0.86‐1.50)
**APACHE II score**	4.77 ± 3.24	10.32 ± 7.11	.001	1.55 (1.11‐1.41)	.039	1.50 (1.02‐2.19)
**Serum MFG‐E8 (ng/mL)**	73.63 ± 121.34	25.45 ± 18.34	<.001	0.65 (0.52‐0.81)	.012	0.51 (0.31‐0.86)

**ABBREVIATIONS**: BMI, Body Mass Index; PLT, Platelets; WBC, White Blood Cell; PT, Prothrombin Time; FIB, Fibrinogen; APTT, Activated Partial Thromboplastin Time; AST, Aspartate aminotransferase; Cr, Creatinine; BUN, Blood Urea Nitrogen; APACHE II, Acute Physiology and Chronic Health Evaluation II; OR, odds ratio; CI, Confidence interval.

### 2.2 MFG‐E8 administration is beneficial in experimental AP

In the mouse model of l‐arginine‐induced AP, serum and pancreatic levels of MFG‐E8 decreased by 65% and 76%, respectively, at 24 h after the induction of AP (Figure S1A,B). Similarly, serum and pancreatic MFG‐E8 levels in l‐arginine‐AP mice began to rise over time after reaching a minimum and eventually returned to almost sham levels 96 h after l‐arginine injection.

**FIGURE 2:**
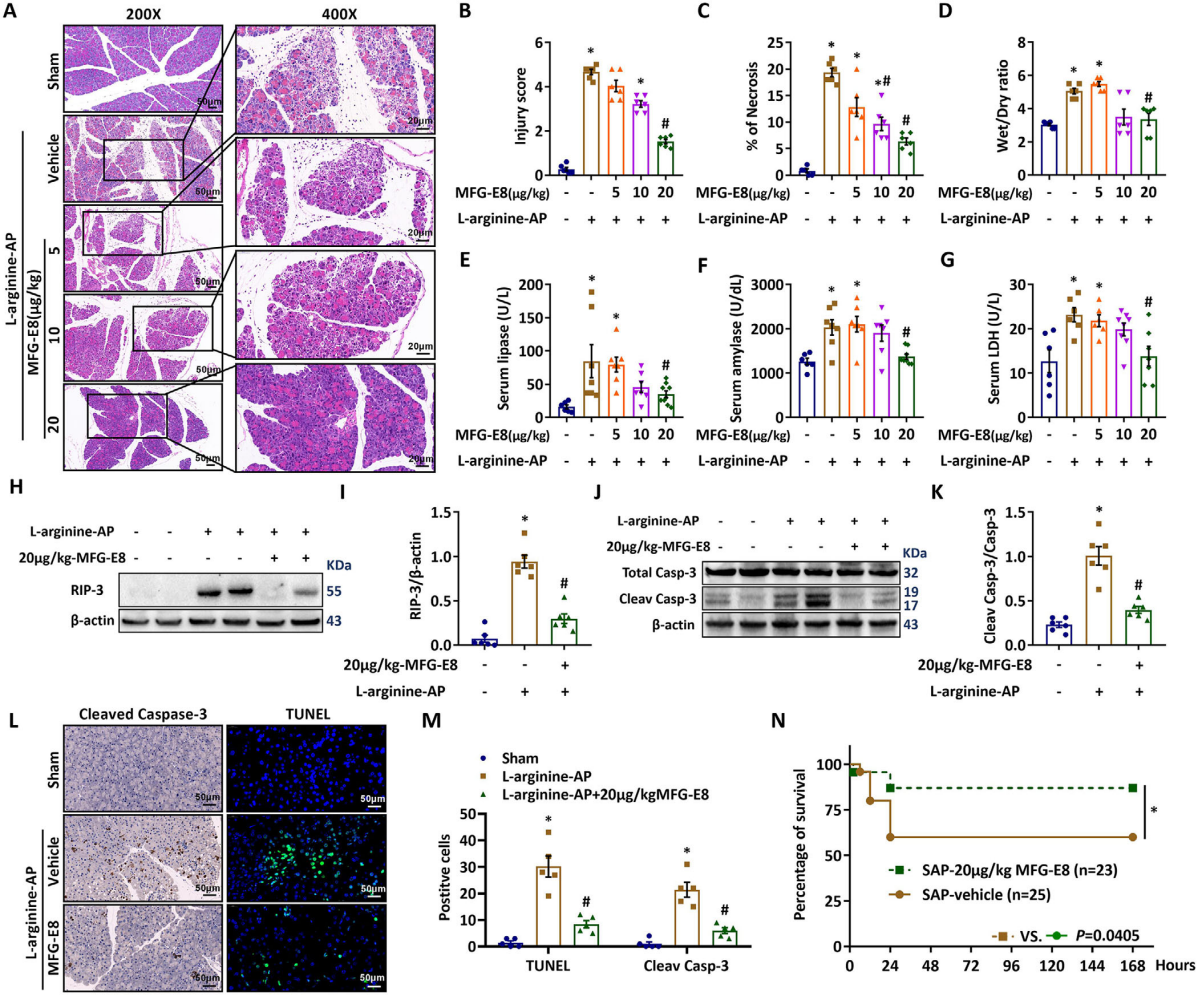
MFG‐E8 administration was protective in experimental acute pancreatitis. In mice, arginine‐AP was induced by 2 hourly intraperitoneal injections of 4.0 g/kg l‐arginine. At 2 h after the last injection of l‐arginine, normal saline (vehicle) or 5, 10, or 20 μg/kg MFG‐E8 was administered through intraperitoneal injection. The animals were sacrificed at 69 h after MFG‐E8 treatment (ie, 72 h after the first injection of l‐arginine). Blood and tissue samples were collected. In additional groups of arginine‐AP mice, the survival was monitored for 168 h after 20 μg/kg‐MFG‐E8 or vehicle treatment. A, Representative photos of hematoxylin and eosin (HE) staining of the pancreas (200× or 400×); B, Pancreatic injury scores; C, Percentages of necrotic areas; D, Pancreatic Wet/Dry ratio; E, Serum lipase levels; F, Serum amylase levels; G, Serum LDH levels; H,I, Western blot analysis of the expression of RIP3 in the pancreas; J,K, Western blot analysis of the expression of caspase 3 in the pancreas; L, Representative photos of cleaved‐caspase 3 and TUNEL staining (400×); M, Quantitative of cleaved‐caspase 3 and TUNEL staining; N, 7‐day survival. n = 4–9/group, error bars indicate the SEM; ^∗^ *P* < .05 versus Sham group; ^#^ *P* < .05 versus Vehicle group. RIP3, receptor‐interacting protein kinase 3; LDH, lactate dehydrogenase; TUNEL, TdT‐mediated dUTP Nick‐End Labeling

To investigate whether MFG‐E8 has any beneficial effects in experimental AP, recombinant MFG‐E8 (5, 10, or 20 μg/kg) was administered 2 h after the second injection of l‐arginine. As shown in Figure 2A,B, l‐arginine injection caused widespread macroscopic injury (average injury score, 5.0 ± 0.3) in the pancreas of vehicle‐treated mice. Approximately 18.7% of acini were nonviable in vehicle‐treated mice (Figure 2C). The wet/dry ratio of the pancreas also increased significantly in vehicle‐treated AP mice (Figure 2D). Consistantly, serum levels of lipase (Figure 2E), amylase (Figure 2F) and LDH (Figure 2G) were also elevated in vehicle‐treated AP mice. Administration of MFG‐E8 improved pancreas architecture (Figure 2A), reduced the pancreas injury score (Figure 2B), lessened nonviable acini (Figure 2C), attenuated pancreatic edema (Figure 2D) and decreased serum levels of lipase (Figure 2E), amylase (Figure 2F) and LDH (Figure 2G). And the beneficial effects of MFG‐E8 appeared to be dose‐dependent. Furthermore, RIP3 expression increased significantly in vehicle‐treated AP mice. Treatment with 20 μg/kg MFG‐E8 reduced RIP3 expression in AP mice (Figure 2H,I). MFG‐E8 facilitates apoptotic cells removal by phagocytes.^21^ As shown in Figure 2J,K, MFG‐E8 treatment significantly reduced cleaved caspase‐3 expression in AP mice (*P* < .05). Consistently, TUNEL staining showed that MFG‐E8 treatment reduced the apoptotic pancreatic cells by 59.3% in AP mice (*P* < 0.05, Figure 2L,M). As shown in Figure 2N, the survival rate in vehicle‐treated AP mice was 80% at 12 h and reduced to 60% at days 1–7. Treatment with 20 μg/kg MFG‐E8 significantly increased the 7‐day survival rate to 91.3% (*P* < .05).

**FIGURE 3:**
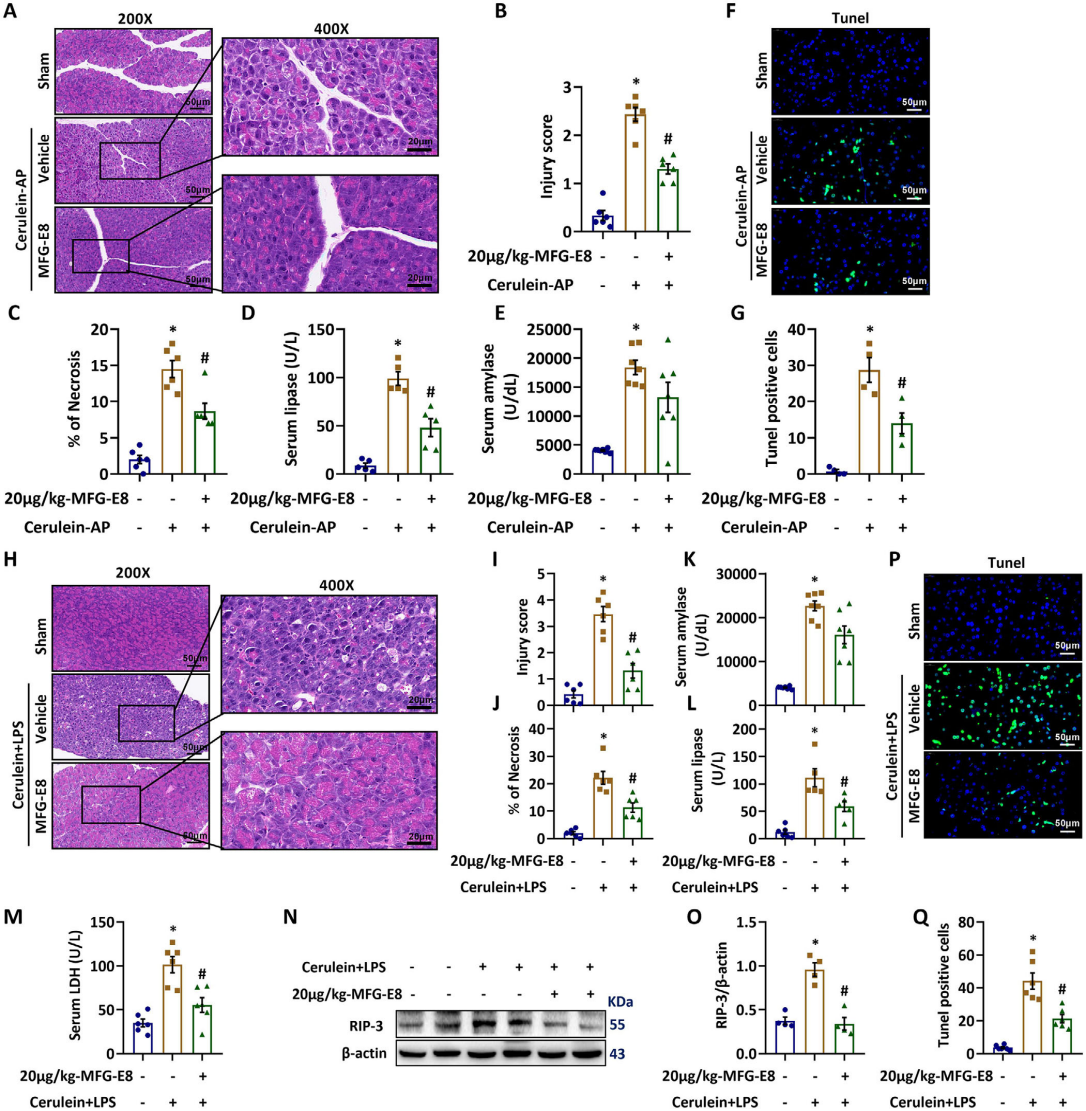
MFG‐E8 administration was protective in cerulein‐ or cerulein + LPS‐induced AP in mice. Cerulein‐AP was induced by 7 hourly injections of 50 μg/kg cerulein. Cerulein + LPS‐AP was induced by 7 hourly injections of 50 μg/kg cerulein. LPS (10 mg/kg) was added to the last cerulein injection. At 30 min after the second injection of cerulein, 20 μg/kg MFG‐E8 were administered through intraperitoneal injection. The animals were sacrificed at 4 h after the last injection of cerulein (ie, 11 h after the first injection of cerulein). Blood and tissue samples were collected. A, Representative photos of hematoxylin and eosin (HE) staining in the pancreas (200× or 400×); B, Pancreatic injury scores; C, Percentages of necrotic areas; D, Serum lipase levels; E, Serum amylase levels; F, Representative photos of TUNEL staining (400×); G, TUNEL positive cell quantitative; H, Representative photos of hematoxylin and eosin (HE) staining in the pancreas (200× or 400×); I, Pancreatic injury scores; J, Percentages of necrotic areas; K, Serum amylase levels; L, Serum lipase levels; M, Serum LDH levels; N,O, Western blot analysis of the expression of RIP3 in the pancreas; P, Representative photos of TUNEL staining (400×); Q, TUNEL positive cell quantitative. n = 4–7/group, error bars indicate the SEM; ^∗^ *P* < .05 versus Sham group; ^#^ *P* < .05 versus Vehicle group. TUNEL, TdT‐mediated dUTP Nick‐End Labeling; LPS, Lipopolysaccharide; RIP3, receptor‐interacting protein kinase 3

The beneficial effects and anti‐apoptosis of exogenous MFG‐E8 administration were also observed in cerulein (Figure 3A‐G) and cerulein+LPS (Figure 3H–Q)‐induced AP in mice.

### 2.3 MFG‐E8 reduces mitochondrial damage in experimental AP

**FIGURE 4:**
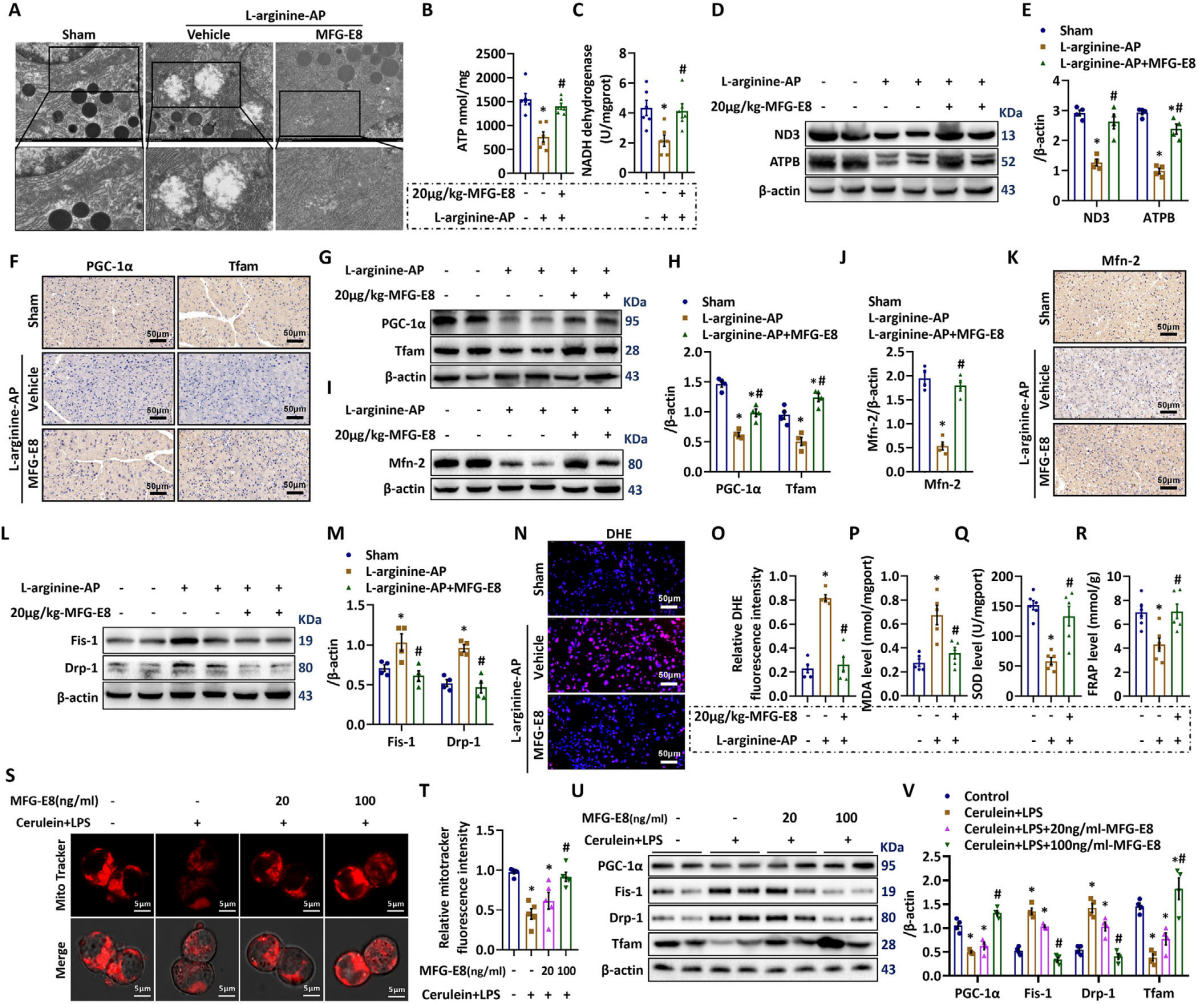
MFG‐E8 improved mitochondrial function and decreased oxidative stress in experimental acute pancreatitis. Arginine‐AP was induced by 2 hourly intraperitoneal injections of 4.0 g/kg l‐arginine. At 2 h after the last injection of l‐arginine, normal saline (vehicle) or 20 μg/kg MFG‐E8 were administered through intraperitoneal injection. The animals were sacrificed at 69 h after MFG‐E8 treatment (ie, 72 h after the first injection of l‐arginine). Blood and tissue samples were collected. Pancreatic AR42J cells (5 × 10^5^/well) were treated with 100 nmol/L cerulein and 10 ng/mL LPS with or without 20 or 100 ng/mL MFG‐E8 for 24 h. A, Ultrastructural alterations in the pancreas (electron microscopy); B, ATP levels in the pancreas; C, NADH dehydrogenase levels in the pancreas; D,E, Western blot analysis of the expression of ND3 and ATPB in the pancreas; F, Representative photos of PGC‐1α and Tfam staining (400×); G,H, Western blot analysis of the expression of PGC‐1α and Tfam in the pancreas; I,J, Western blot analysis of the expression of Mfn‐2 in the pancreas; K, Representative photos of Mfn‐2 staining (400×); L,M, Western blot analysis of the expression of Fis‐1 and Drp‐1 in the pancreas; N, Representative images of DHE fluorescence staining in the pancreas (400×); O, Relative fluorescence intensity of DHE staining; P, MDA levels in the pancreatic tissue; Q, SOD values in the pancreatic tissue; R, FRAP levels in the pancreatic tissue; S,T, Representative images of immunofluorescence staining of MitoTracker red (1500×) and relative fluorescence intensity of MitoTracker red in AR42J cells; U,V, Western blot analysis of the expression of PGC‐1α, Tfam, fis‐1, and Drp‐1 in AR42J cells. n = 4–6/group, error bars indicate the SEM; ^∗^ *P* < .05 versus Sham group or versus control; ^#^ *P* < .05 versus Vehicle group or versus cerulein + LPS group. PGC‐1α, peroxisome proliferative activated receptor‐γ coactivator 1α; Tfam, mitochondrial transcription factor; Mfn‐2, Mitofusin‐2; ND3, NADH dehydrogenase subunit 3; DHE, Dihydroethidium; MDA, manoldialdehyde; SOD, superoxide dismutase; FRAP, Ferric Reducing Antioxidant Power

Mitochondrial damage is a key step in the pathogenesis of AP. Consistent with our previous findings, abnormal swollen mitochondria with disrupted cristae were frequently observed in vehicle‐treated AP mice. MFG‐E8 treatment led to significant improvements in mitochondrial ultrastructure in AP mice (Figure 4A). A major function of mitochondria is to produce ATP. As shown in Figure 4B, l‐arginine injection reduced pancreatic mitochondrial ATP levels by 51.6%. Administration of 20 μg/kg MFG‐E8 increased mitochondrial ATP levels by 94.7% (P < 0.05). Reduced NADH dehydrogenase levels often indicate impaired mitochondrial function.^22^ NADH dehydrogenase activities in vehicle‐treated AP mice were 59.3% lower than those in control mice. Administration of MFG‐E8 almost restored NADH dehydrogenase activities to sham levels (*P* < .05, Figure 4C). The levels of NADH dehydrogenase 3 (ND3) protein showed comparable results (Figure 4D,E). The expression of ATPB, another important player in the mitochondrial respiratory chain, also had similar changes (Figure 3D,E).

Healthy mitochondrial dynamics, including mitochondrial biogenesis, fusion and fission, are vital for a proper mitochondrial function. PGC‐1α and Tfam are important regulators of mitochondrial biogenesis.^23^ Both immunohistochemical staining (Figure 4F) and Western blot analysis (Figure 4G,H) revealed that PGC‐1α and Tfam were significantly downregulated in vehicle‐treated AP mice. MFG‐E8 treatment markedly increased PGC‐1α and Tfam levels in AP mice. Mfn‐2 regulates mitochondrial fusion, while Drp‐1 and Fis‐1 regulate mitochondrial fission.^24^ Immunohistochemical staining and western blot analysis (Figure 4I‐K) showed Mfn‐2 was downregulated in vehicle‐treated AP mice. Western blot analysis (Figure 4L,M) revealed that Drp‐1 and Fis‐1 were upregulated after the induction of AP. MFG‐E8 treatment increasd Mfn‐2 expression and decreasd Drp‐1 and Fis‐1 expression. Thus, MFG‐E8 treatment improved mitochondrial biogenesis, increased mitochondrial fusion and inhibited mitochondrial fissure in AP.

Mitochondrial dysfunction can lead to oxidative stress.^25^ DHE staining detects reactive oxygen species (ROS). As shown in Figure 4N,O, fluorescence intensity of DHE increased significantly in vehicle‐treated AP mice. MFG‐E8 treatment decreased the fluorescence intensity of DHE by 47.2%. Consistently, pancreatic levels of manoldialdehyde (MDA, Figure 4P) were significantly increased, while superoxide dismutase (SOD, Figure 4Q) and Ferric Reducing Antioxidant Power (FRAP, Figure 4R) were markedly decreased after the induction of AP. MFG‐E8 treatment decreased MDA and increased SOD and FRAP in AP mice.

To explore MFG‐E8's direct effect on mitochondrial function in pancreatic acinus cells, Pancretic AR42J cells were treated with cerulein and LPS. MitoTracker stains mitochondria in live cells. As shown in Figure 4S,T, cerulein + LPS decreased MitoTracker staining in cultured AR42J cells. MFG‐E8 dose‐dependently increased mitoTracker fluorescence intensity in cerulein + LPS‐treated acinar cells (*P* < .05). Consistent with the above‐mentioned in vivo findings, MFG‐E8 also increased PGC‐1α and Tfam levels and decreased Drp‐1 and Fis‐1 levels in cultured AR42J cells (Figure 4U,V).

### 2.4 MFG‐E8 deficiency aggravated pancreatic injury in AP mice

**FIGURE 5:**
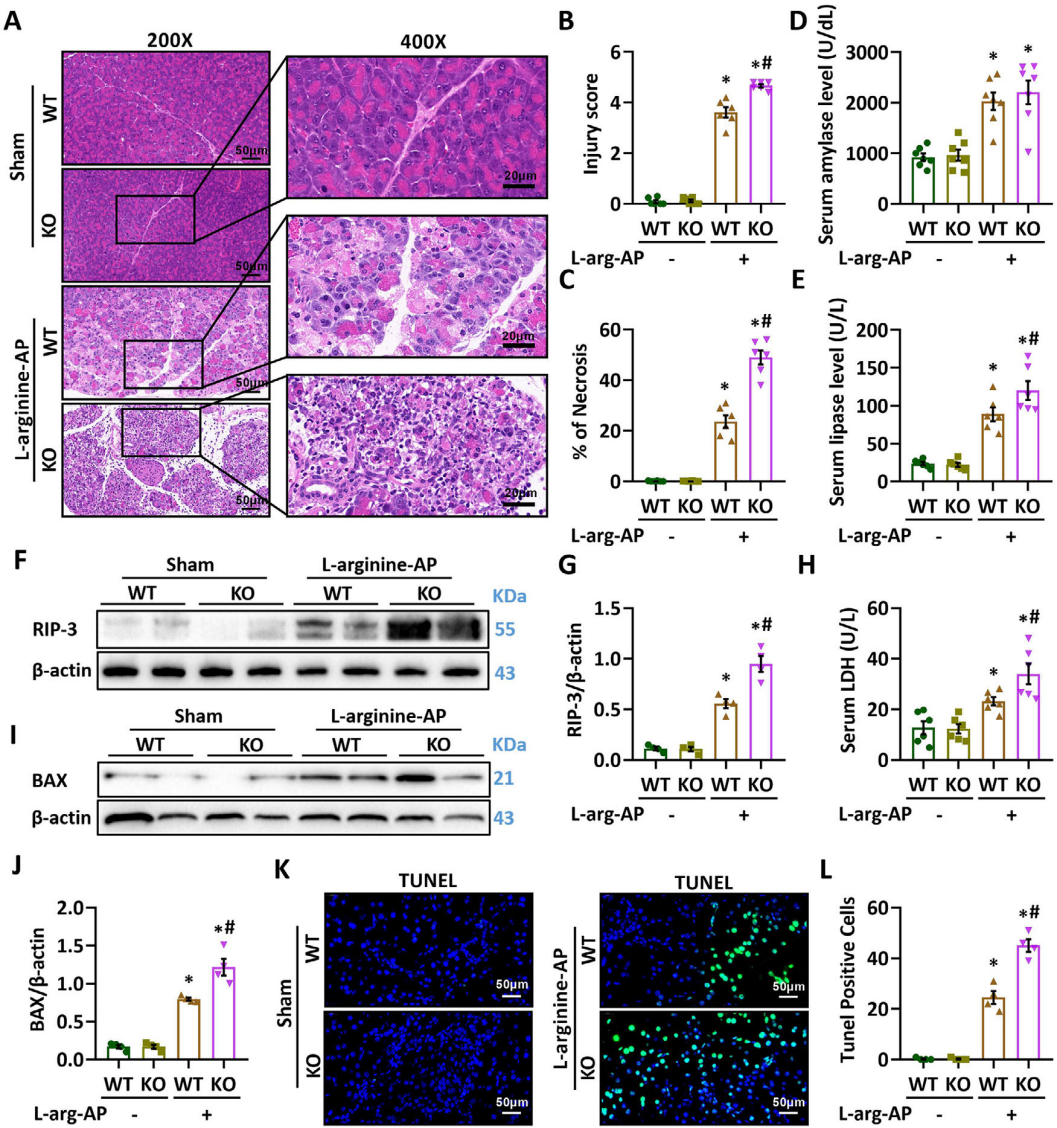
Deletion of MFG‐E8 aggravated pancreatic injury in AP mice. Arginine‐AP was induced by 2 hourly intraperitoneal injections of 4.0 g/kg l‐arginine. The animals were sacrificed at 69 h after MFG‐E8 treatment (ie, 72 h after the first injection of l‐arginine). A, Representative photos of hematoxylin and eosin (HE) staining in the pancreas (200× or 400×); B, Pancreatic injury scores; C, percentages of necrotic areas; D, Serum amylase levels; E, serum lipase levels; F,G, Western blot analysis of the expression of RIP3 in the pancreas; H, Serum LDH levels; I,J, Western blot analysis of the expression of BAX in the pancreas; K,L, Representative photos of TUNEL staining (400×) and quantitative of TUNEL staining. n = 4–7/group, error bars indicate the SEM; ^∗^ *P* < .05 versus Sham group; ^#^ *P* < .05 versus Vehicle group. RIP3, receptor‐interacting protein kinase 3; LDH, lactate dehydrogenase; TUNEL, TdT‐mediated dUTP Nick‐End Labeling

**FIGURE 6:**
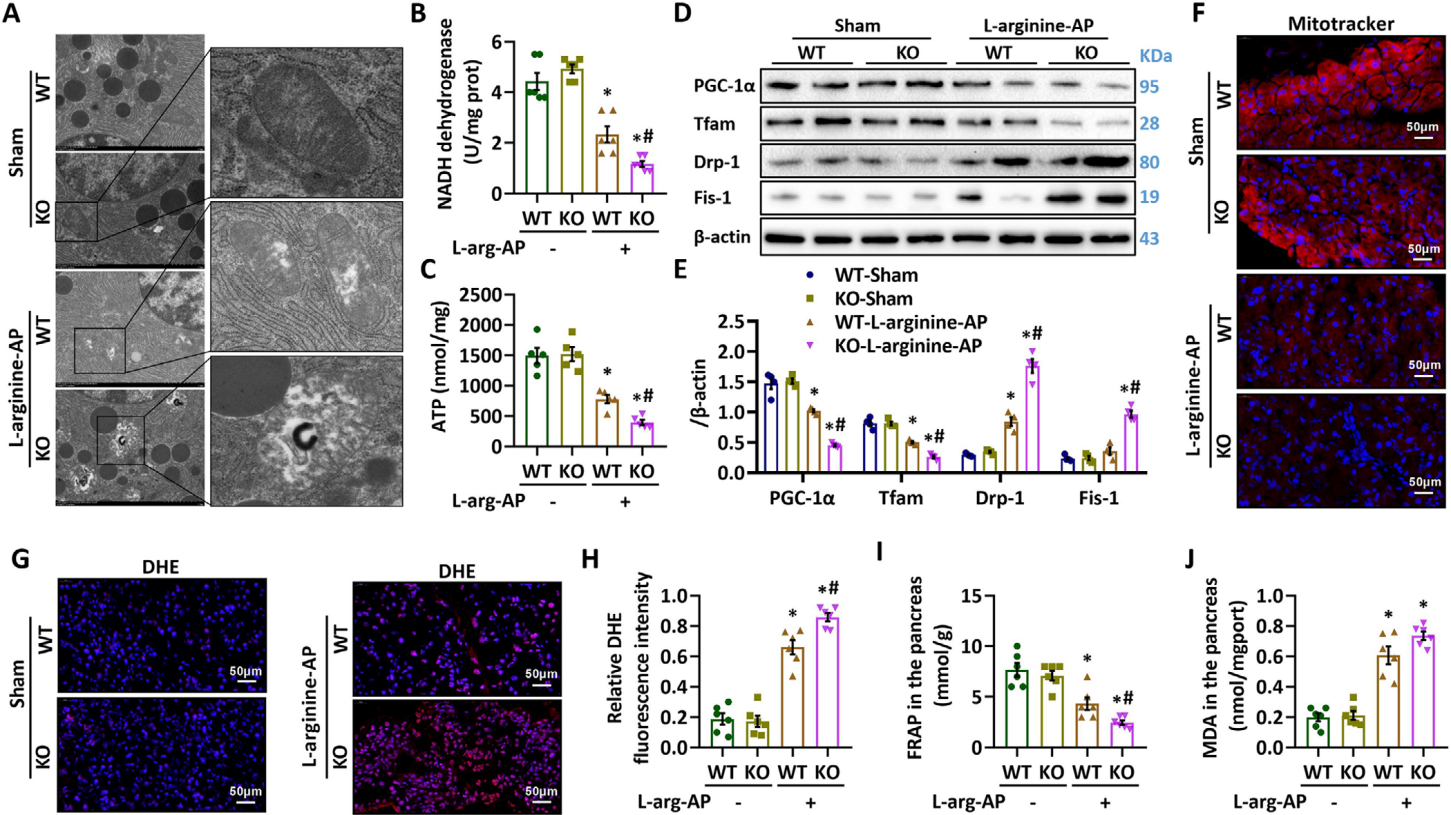
Deletion of MFG‐E8 exaggerated pancreatic mitochondrial dysfunction and increased oxidative stress in AP mice. Arginine‐AP was induced by 2 hourly intraperitoneal injections of 4.0 g/kg l‐arginine. The animals were sacrificed at 69 h after MFG‐E8 treatment (ie, 72 h after the first injection of l‐arginine). A, Ultrastructural alterations in the pancreas (electron microscopy); B, NADH dehydrogenase levels in the pancreas; C, ATP levels in the pancreas; D,E, Western blot analysis of the expression of PGC‐1α, Tfam, fis‐1, and Drp‐1 in the pancreas; F, Representative images of Mitotracker fluorescence staining in the pancreas (400×); G, Representative images of DHE fluorescence staining in the pancreas (400×); H, Relative fluorescence intensity of DHE staining; I, FRAP values in the pancreatic tissue; J, MDA level in the pancreatic tissue. *n* = 4–7/group, error bars indicate the SEM; ^∗^ *P* < .05 versus Sham group; ^#^ *P* < .05 versus Vehicle group. PGC‐1α, peroxisome proliferative activated receptor‐γ coactivator 1α; Tfam, mitochondrial transcription factor; Mfn‐2, Mitofusin‐2; DHE, Dihydroethidium; MDA, manoldialdehyde; FRAP, ferric reducing antioxidant power

To study MFG‐E8's contribution in the pathogenesis of AP, we examined l‐arginine injection‐induced AP in MFG‐E8‐deficient mice. As shown in Figure 5A,B, MFG‐E8 KO mice displayed more severe pancreatic injury after l‐arginine injection than WT mice. The MFG‐E8 KO mice also had larger necrotic areas and higher levels of RIP‐3 expression than WT mice (Figures 5C‐H). Apoptosis was also further increased in MFG‐E8 KO mice after l‐arginine injection (Figures 5I‐L). This potentiated pancreatic injury was associated with more serious mitochondrial damage (Figures 6A‐F) and exaggerated oxidative stress (Figure 6G‐J) than WT mice after l‐arginine injection.

### 2.5 MFG‐E8 activates the integrin‐FAK‐STAT3 signaling pathway in AP

**FIGURE 7:**
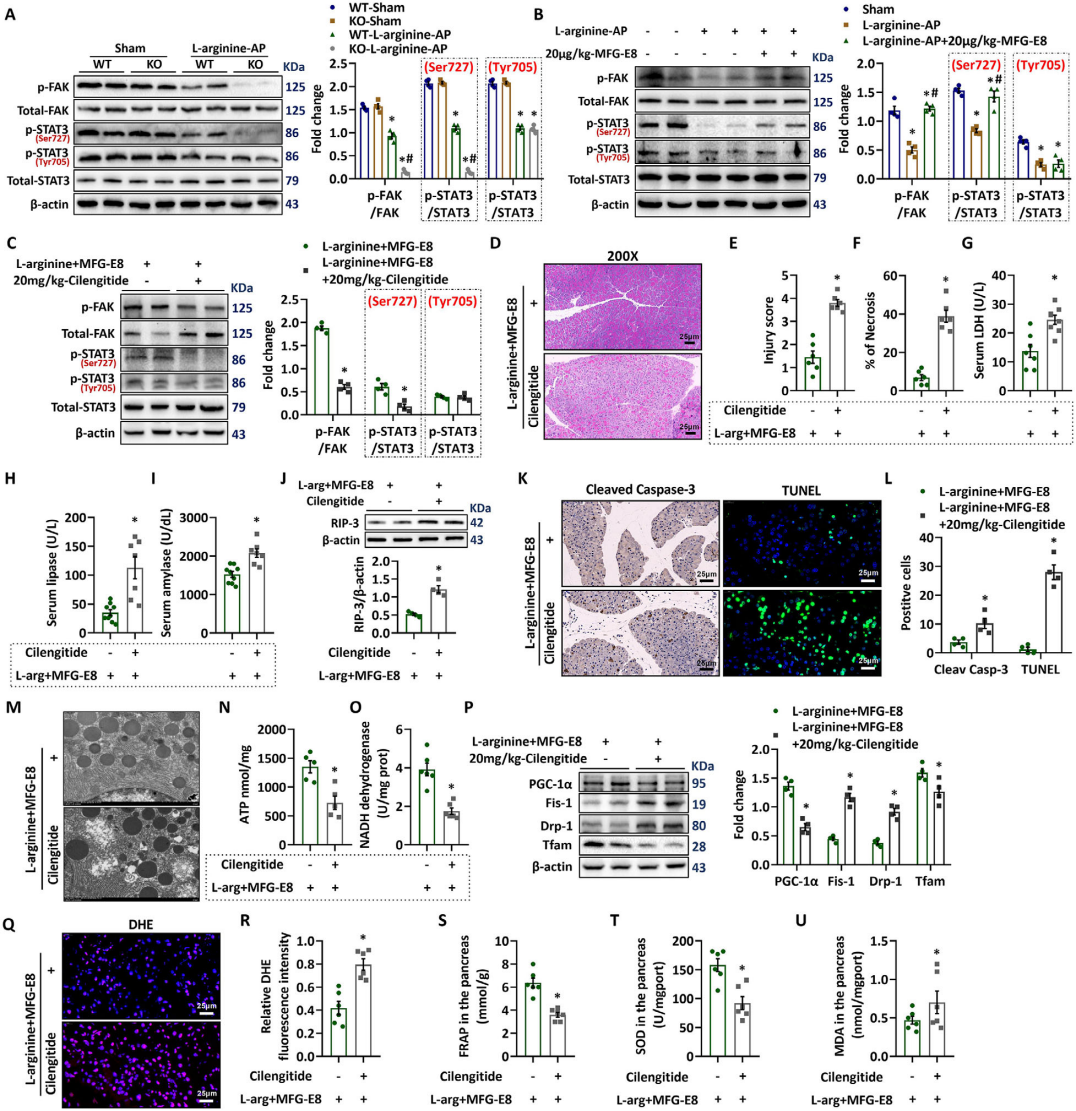
MFG‐E8 activated the FAK‐STAT3 signaling pathway through integrin αVβ3/5 in AP mice. Arginine‐AP was induced by 2 hourly intraperitoneal injections of 4.0 g/kg l‐arginine. At 2 h after the last injection of l‐arginine, normal saline (vehicle), or 20 μg/kg MFG‐E8 were administered through intraperitoneal injection. To determine whether the protective effect of MFG‐E8 in AP is mediated through integrin αVβ3/5, 20 mg/kg cilengitide, a specific integrin αVβ3/5 antagonist, was administered intraperitoneally at 1 h before the injection of MFG‐E8 (ie, 1 h after the last injection of l‐arginine). The animals were sacrificed at 69 h after MFG‐E8 treatment (ie, 72 h after the first injection of l‐arginine). A‐C, Western blot analysis of the expression of FAK and STAT3 in the pancreas; D, Representative photos of hematoxylin and eosin (HE) staining in the pancreas (200×); E, Pancreatic injury scores; F, Percentages of necrotic areas; G, Serum LDH levels; H, Serum lipase levels; I, Serum amylase levels; J, Western blot analysis of the expression of RIP3 in the pancreas; K, Representative photos of cleaved‐caspase 3 and TUNEL staining (400×); L, Quantitative of cleaved‐caspase 3 and TUNEL staining; M,Ultrastructural alterations in the pancreas (electron microscopy); N, ATP levels in the pancreas; O, NADH dehydrogenase levels in the pancreas; P, Western blot analysis of the expression of PGC‐1α, Tfam, fis‐1, and Drp‐1 in the pancreas; Q, Representative images of DHE fluorescence staining in the pancreas (400×); R, Relative fluorescence intensity of DHE staining; S, FRAP values in the pancreatic tissue; T, SOD levels in the pancreatic tissue; U, MDA levels in the pancreatic tissue. n = 4–9/group, error bars indicate the SEM; ^∗^ *P* < .05 versus Sham group; ^#^ *P* < .05 versus Vehicle group. RIP3, receptor‐interacting protein kinase 3; LDH, lactate dehydrogenase; TUNEL, TdT‐mediated dUTP Nick‐End Labeling; PGC‐1α, peroxisome proliferative activated receptor‐γ coactivator 1α; Tfam, mitochondrial transcription factor; Mfn‐2, Mitofusin‐2; DHE, Dihydroethidium; MDA, manoldialdehyde; SOD, superoxide dismutase; FRAP, Ferric Reducing Antioxidant Power

MFG‐E8 can bind to αvβ3 and αvβ5 integrins.^17^, ^26^ To explore the possible mechanism responsible for MFG‐E8's beneficial effects in AP, we first measured the levels of phosphorylated FAK and STAT3. As shown in Figure 7A, AP was associated with decreased phosphorylation of FAK and STAT3. And MFG‐E8 KO mice displayed even lower phosphorylation of FAK and STAT3 than WT mice after L‐arginine injection. Recombinant MFG‐E8 treatment increased the levels of phosphorylated FAK and STAT3 to almost sham levels (Figure 7B).

Administration of cilengitide, a specific inhibitor of αvβ3/5 integrin, 1 h before MFG‐E8 injection effectively blocked MFG‐E8‐induced phosphorylation of FAK and STAT3 in AP mice (Figure 7C). And cilengitide also blocked the effects of MFG‐E8 on pancreatic injury (Figure 7D‐J), apoptosis (Figure 7K‐L) in AP mice. The effects of MFG‐E8 on mitochondrial function (Figure 7 M‐P) and oxidative stress (Figure 7Q‐U) were also eliminated by the administration of cilengitide in AP mice.

**FIGURE 8:**
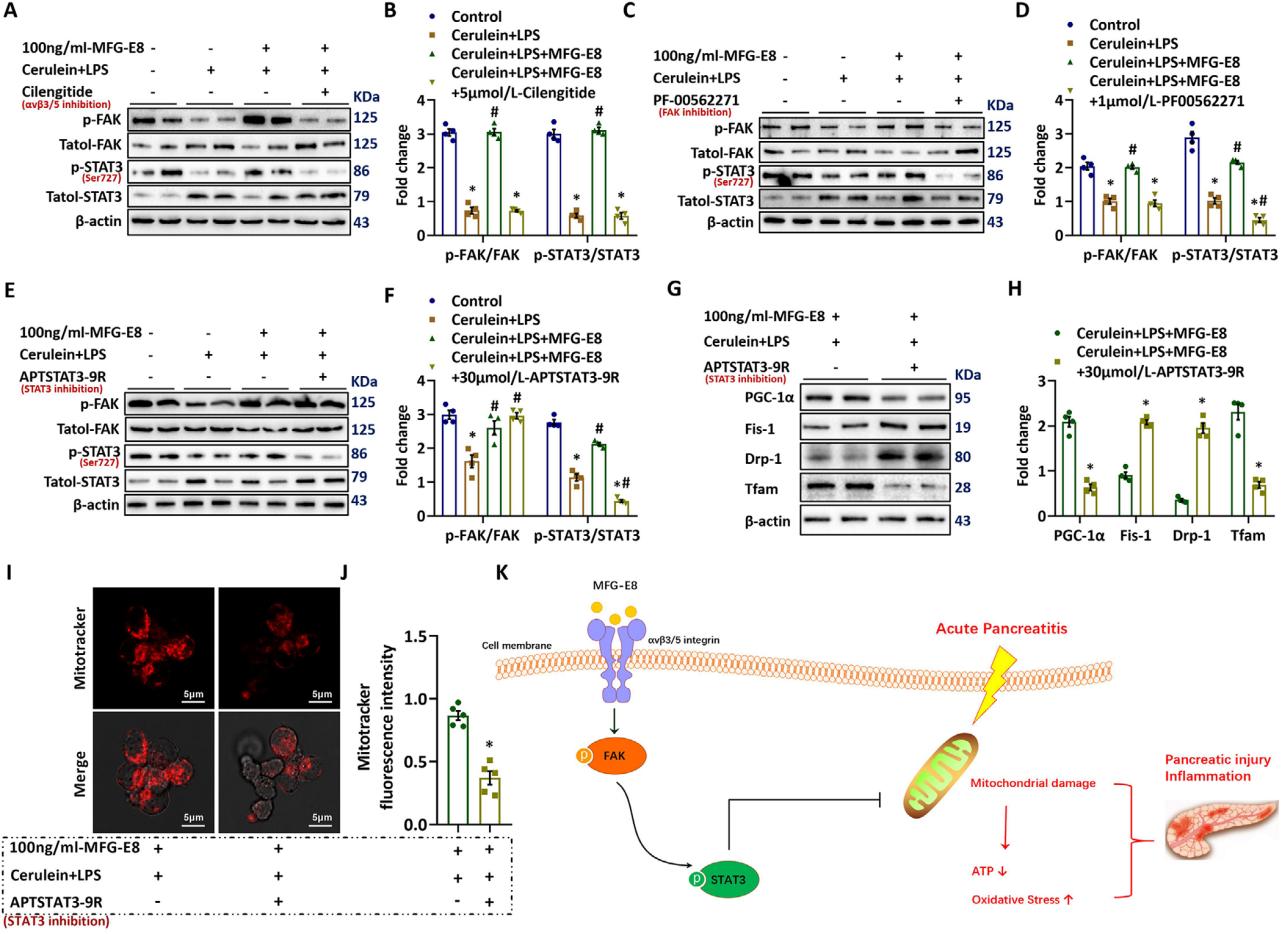
MFG‐E8 activated the FAK‐STAT3 (ser727) signaling pathway in pancreatic cells. Pancreatic AR42J cells (5 × 10^5^/well) were treated with 100 nmol/L cerulein and 10 ng/mL LPS with or without 100 ng/mL MFG‐E8 for 24 h. To determine whether the protective effect of MFG‐E8 in AP is mediated through integrin αVβ3/5‐FAK‐STAT3 (ser727), 1 μmol/L‐PF‐00562271, a specific FAK antagonist, 30 μmol/L‐APTSTAT3‐9R, a specific STAT3 antagonist, was administered with MFG‐E8, respectively. A‐F, Western blot analysis of the expression of FAK and STAT3 (ser727) in AR42J cells; G,H, Western blot analysis of the expression of PGC‐1α, Tfam, fis‐1, and Drp‐1 in AR42J cells; I,J, Representative images of immunofluorescence staining of MitoTracker red (1500×) and relative fluorescence intensity of MitoTracker red in AR42J cells; K, Schematic diagram showing MFG‐E8 protects against AP through activation of the integrin‐FAK‐STAT3 (ser727) signaling pathway. n = 4–5, error bars indicate the SEM. ^∗^ *P* < .05 versus control; ^#^ *P* < .05 versus cerulein + LPS group. PGC‐1α, peroxisome proliferative activated receptor‐γ coactivator 1α; Tfam, mitochondrial transcription factor; Mfn‐2, Mitofusin‐2

To investigate the downstream signaling of MFG‐E8 in pancreatic acinus cells, specific inhibitors for αvβ3/5 integrin, FAK and STAT3 were applied respectively. Administration of cilengitide blocked MFG‐E8‐induced phosphorylation of FAK and STAT3 in cerulein + LPS‐treated pancreatic acinus cells (Figure 8A,B). The addition of PF‐00562271, a specific FAK inhibitor, blocked MFG‐E8‐induced phosphorylation of both FAK and STAT3 in cerulein + LPS‐treated pancreatic acinus cells (Figure 8C,D). However, the specific STAT3 inhibitor, APTSTAT3‐9R, only blocked MFG‐E8‐induced phosphorylation of STAT3, but not FAK in cerulein + LPS‐treated pancreatic acinus cells (Figure 8E,F). APTSTAT3‐9R also blocked MFG‐E8's effect on the expression of PGC‐1α, Tfam, Drp‐1, Fis‐1, and mitotracker staining (Figure 8G‐J).

Thus, MFG‐E8 administration protects against AP possibly by restoring mitochondrial function via activation of the integrin‐FAK‐STAT3 signaling pathway (Figure 8K).

## 3 DISCUSSION

Here, we showed that serum MFG‐E8 concentrations were inversely correlated with clinical severity of AP patients and treatment with recombinant MFG‐E8 mitigated pancreatic injury and decreased mortality in l‐arginine‐induced AP, suggesting MFG‐E8 is an endogenous protective mediator in AP. MFG‐E8 KO mice display an exaggerated pancreatic injury after induction of AP, providing additional evidence for MFG‐E8's protective role in AP.

MFG‐E8 is a secreted protein. However, the main source of MFG‐E8 in the circulation remains to be determined. Although MFG‐E8 was originally identified in the lactating mammary glands, it is detectable in various tissues including the pancreas.^27^, ^28^, ^29^, ^30^, ^31^ AP is an inflammatory condition and often associated with systemic inflammation. Serum MFG‐E8 levels have been shown to be downregulated in acute inflammatory conditions such as sepsis and ischemia‐reperfusion injury.^32^, ^33^, ^34^ Here, we showed that serum concentrations of MFG‐E8 and CRP were negatively correlated in AP patients, suggesting an inverse relationship between serum MFG‐E8 concentrations and inflammatory severity. Consistent with the findings in the current study, MFG‐E8 treatment decreased inflammatory responses and improved organ function in rodent models of sepsis.^35^, ^36^ Therefore, severe systemic inflammation may be the reason for the decreased MFG‐E8 levels in AP. The increase in serum MFG‐E8 concentrations at later time points may be related to the reduced inflammatory response at the recovery stage of AP.

Mitochondrial dysfunction contributes significantly to the pathogenesis of AP.^3^ Mitochondria are vital for the physiological and pathophysiologic functions of the pancreas. Impaired mitochondrial integrity predisposes pancreatic cells to energy depletion and free radical generation. Mitochondrial damage can lead to the development of hyperamylasemia, trypsinogen activation, necrosis, and inflammation in AP.^10^ Restoring mitochondrial function, therefore, offers a potential treatment option for AP.^12^ Here, we showed that MFG‐E8 administration protected mitochondrial ultrastructure and upregulated PGC‐1a, Tfam, ND3, and ATPB levels in AP mice, suggesting reduced mitochondrial damage, enhanced mitochondrial biogenesis, and improved mitochondrial function after MFG‐E8 treatment. MFG‐E8‐deficient mice, on the other hand, suffered greater mitochondrial damage after l‐arginine administration. Thus, MFG‐E8 may be of great importance in maintaining a proper mitochondrial function in AP.

Many of MFG‐E8's biological functions are mediated via binding to the αvβ3/5 integrin.^37^, ^38^, ^39^, ^40^ The αvβ3/5 integrin is the only known receptor of MFG‐E8. Although there has been no report on MFG‐E8's effects on mitochondrial function, a recent study has shown that activation of the integrin‐FAK‐STAT3 signaling pathway promotes the repair of damaged mitochondria.^19^ Here, we found MFG‐E8 increased phosphorylation of FAK and STAT3. STAT3 is an important regulator of mitochondrial function. The phosphorylation of STAT3 at S727 can stimulate mitochondrial bioenergetic function by binding to prohibitin 1,^41^ decrease ROS production by facilitating complex I coupling,^42^ and maintain mitochondrial membrane potential by interacting with cyclophilin D.^43^ The experiments with antagonists showed that cilengitide, a specific αvβ3 and αvβ5 integrin inhibitor, abolished MFG‐E8's beneficial effects in AP. APTSTAT3‐9R, a specific STAT3 antagonist, also eliminated MFG‐E8's beneficial effects under such a condition. PF00562271, a specific FAK inhibitor, blocked MFG‐E8‐induced STAT3 phosphorylation. Thus, MFG‐E8 might activate the FAK‐STAT3 pathway via binding to αvβ3/5 integrins.

Oxidative stress is a major factor that causes pancreatic injury in AP.^44^ Excessive ROS production not only causes direct cellular damage but also activates a cascade of inflammatory mediators, which exaggerates tissue injury.^45^ Suppressing oxidative stress by either providing exogenous antioxidants or activating the endogenous anti‐oxidative mechanism has been shown to be protective in experimental AP.^46^, ^47^, ^48^, ^49^ Mitochondrial injury is closely related to oxidative stress.^50^ Mitochondrial dysfunction not only results in augmented production of ROS, but also hampers the cellular antioxidant defense. Here, we showed that MFG‐E8 administration inhibited oxidative stress in AP mice. In this regard, inhibition of oxidative stress via improving mitochondrial function may contribute to MFG‐E8's protective effects in experimental AP.

MFG‐E8 enables apoptotic cells removal by phagocytes.^14^, ^36^, ^51^ It facilitates the interaction between the exposed phosphatidylserine (PS) on apoptotic cells and integrin expressed on the phagocytes. Inadequate removal of apoptotic cells is considered to be a major mechanism for the development of autoimmune diseases in MFG‐E8 KO mice.^52^ Both apoptosis and necrosis are implicated in the pathogenesis of AP.^9^ Several clinical studies have shown that patients with mild AP often have extensive apoptosis, while patients with severe AP commonly have extensive necrosis.^53^, ^54^ In animal studies, the induction of apoptosis has been shown to be protective in AP.^53^ Thus, apoptosis is proposed as a favorable response in the development of AP. However, the underlying mechanism remains unknown. Here, we found that MFG‐E8 treatment decreased both apoptosis and necrosis after AP, whereas the accumulation of apoptotic cells in the pancreas of MFG‐E8 deficient mice after L‐arginine administration did not result in reduced pancreatic damage. These results suggest that induction of apoptosis per se is not crucial for mitigating pancreatic injury under such conditions. The removal of apoptotic cells by macrophages can trigger an anti‐inflammatory response.^55^ In this regard, the subsequent removal of apoptotic cells may be the real reason behind the beneficial effect of apoptosis induction in AP. In addition, the protection of mitochondrial function by MFG‐E8 prevents cell death, diminishes the release of DAMPs, and therefore lowers the activation of an inflammatory response.

There are some limitations in this study. The model of l‐arginine‐induced AP is widely used to study novel therapeutics due to its severity, which mimics that of bile salt‐induced pancreatitis under specific conditions.^56^ Moreover, we demonstrated the protective effects of MFG‐E8 not only in l‐arginine‐induced AP, but also in cerulein‐ and cerulein+LPS‐induced AP, suggesting MFG‐E8 is a promising candidate for the treatment for AP. However, the efficacy of MFG‐E8 in other models of AP remains to be determined. In the current study, we showed that selective inhibition of αvβ3/5 integrin blocked MFG‐E8's beneficial effects in cerulein + LPS‐treated pancreatic acinus cells, suggesting the pancreatic acinus cell is a target of MFG‐E8 in AP. However, the effects of MFG‐E8 on other αvβ3/5 integrin‐expressing cells may also contribute to MFG‐E8's protection in AP. Since chronic pancreatitis is often developed in patients with acute pancreatitis, it is highly possible that MFG‐E8 may also be protective in chronic pancreatitis. However, the exact effect and detailed mechanism of MFG‐E8 in chronic pancreatitis warrant further investigation. In our previous study, we found that mice have significant mitochondrial dysfunction in the pancreas at 24 h after l‐arginine injection, while their pancreatic injury peaks at 72 h.^12^ Therefore, we speculated that administration of MFG‐E8 within 24 h would have some degrees of protection in AP. However, the time‐course of MFG‐E8 treatment in AP needs further investigation.

In conclusion, serum MFG‐E8 concentrations are decreased in AP patients and administration of recombinant MFG‐E8 is beneficial in experimental AP. MFG‐E8 deficiency leads to exaggerated pancreatic damage in experimental AP. MFG‐E8's beneficial effect in AP is associated with improvement in mitochondrial function via activation of the integrin‐FAK‐STAT3 signaling pathway in the pancreas. Thus, MFG‐E8 appears to be an endogenous protective mediator in the pathogenesis of AP. Targeting the action of MFG‐E8 may present a potential therapeutic option for AP patients.

## 4 MATERIALS AND METHODS

### 4.1 Study design

We intended to investigate the changes of serum MFG‐E8 concentrations in AP patients and the effect of recombinant MFG‐E8 treatment in experimental AP. For the clinical study, blood samples were acquired from healthy controls and AP patients recruited to the the First Affiliated Hospital of the Xi'an Jiaotong University, and their serum MFG‐E8 concentrations were assessed by ELISA. Sample sizes were based on availability. Since this is a noninterventional study, no blinding or randomization was applied. For the preclinical study, animals were assigned to different experimental groups randomly. The outcomes included pancreatic injury, blood concentrations of pancreas‐derived enzymes, and survival rates.

### 4.2 Patients

One hundred thirty‐four AP patients (age ≥ 18 years) who were treated at the First Affiliated Hospital of the Xi'an Jiaotong University from Jan 2018 to Jan 2019 were included in this study. AP was diagnosed according to International Atlanta Symposium on Acute Pancreatitis.^57^ Sixty‐nine healthy volunteers were recruited as healthy controls. The study was approved by the hospital's Ethics Committee. All study participants signed the informed consent.

### 4.3 Experimental animals

Male adult C57BL/6 J mice (Experimental Animal Center of Xi'an Jiaotong University, Xi'an, China) and MFG‐E8 knockout (KO) mice (Shanghai Model Organisms Center, Inc., Shanghai, China) were used in this study. The MFG‐E8 KO mice were generated by deleting exons 2–6 of the mfg‐e8 gene on a C57BL/6 J background using the CRISPR/Cas9 technique. The mice were fed on a standard laboratory chow diet and housed in a temperature‐controlled room on a 12‐h light/dark cycle. The study protocol was approved by the Institutional Animal Care and Use Committee of the Ethics Committee of Xi'an Jiaotong University Health Science Center.

### 4.4 Experimental models

The mice (8‐10 weeks, 20–22 g) were fasted for 12 h prior to the procedure. Arginine‐AP was induced by 2 hourly intraperitoneal injections of 4.0 g/kg l‐arginine (Sigma‐Aldrich, Shanghai, China). Two hours after the last injection of L‐arginine, normal saline (vehicle) or 5, 10, or 20 μg/kg MFG‐E8 (RD System, Inc. Minnesota, USA) were administered intraperitoneally. The doses of MFG‐E8 used in this study were chosen on the basis of previous publications in sepsis and ischemia‐reperfusion injury.^32^, ^35^ The mice were anesthetized with isoflurane inhalation at 72 h after the first injection of l‐arginine (ie, 69 h after MFG‐E8 treatment). Blood samples and pancreatic tissues were harvested. To determine the role of αvβ3/5 integrin in MFG‐E8's effect in AP, cilengitide (20 mg/kg, SELLECK, Texas, USA), a specific αvβ3/5 integrin inhibitor, were administered through intraperitoneal injection at 1 h after the last injection of l‐arginine. The animals were euthanized at 72 h after the first injection of l‐arginine to harvest blood and tissue samples. In additional groups of l‐arginine‐AP mice, the survival was monitored for 7 days after MFG‐E8 or vehicle treatment.

Cerulein‐AP was induced by 7 hourly injections of cerulein (50 μg/kg, Solarbio, Beijing, China). At 30 min after the second injection of cerulein, 20 μg/kg MFG‐E8 was administered intraperitoneally. Blood and tissue samples were harvested at 11 h after the first injection of cerulein (ie, 4 h after the last injection of cerulein).

Cerulein + LPS‐AP was induced by 7 hourly injections of cerulein (50 μg/kg). Lipopolysaccharide (LPS, 10 mg/kg, L8880, Solarbio, Beijing, China) was added to the last cerulein injection.^58^ At 30 min after the second injection of cerulein, 20 μg/kg MFG‐E8 were administered intraperitoneally. Blood and tissue samples were harvested at 4 h after the last injection of cerulein (ie, 11 h after the first injection of cerulein).

### 4.5 Cell culture

Pancreatic AR42J cells (zq0145, ScienCell Research Laboratories, Inc., Shanghai, China) were cultured in Ham's F‐12K medium (Procell Life Science&Technology Co., Ltd., Wuhan, China) with 20% fetal bovine serum in a humidified incubator at 37°C with 5% CO_2_^58^. AR42J cells (5 × 10^5^/well) were plated into six‐well culture plates for 24 h. Then, the cells were treated with 100 nmol/L cerulein (C6660, Solarbio, USA) and 10 ng/mL lipopolysaccharide (LPS; L‐8880, Solarbio, USA) with or without 20 or 100 ng/mL MFG‐E8 (2805‐MF, RD System, Inc. Minnesota, USA) for 24 h. In additional groups of AR42J cells, PF00562271 (S2672, SELLECK, Texas, USA), a specific FAK inhibitor or APTSTAT3‐9R (S8197, SELLECK, Texas, USA), a specific STAT3 antagonist, were administered together with 100 ng/mL MFG‐E8.

### 4.6 Immunohistochemical Staining

Immunohistochemical staining was performed as we described before.^59^ The primary antibodies are caspase‐3 (ab13847, rabbit polyclonal, Abcam, USA), PGC‐1α (ab54481, antibody, Abcam, USA), Tfam (ab272885, antibody, Abcam, USA) and Mfn‐2 (ab56889, antibody, Abcam, USA).

### 4.7 Histologic evaluation

Pancreatic tissue sections were stained with hematoxylin and eosin (H&E). The pancreatic tissue damage was assessed using Schmidt's histological scoring system.^60^

### 4.8 Transmission electron microscopy

Seventy nm ultra‐thin sections of pancreatic samples were stained with uranyl acetate and lead citrate. Pancreatic ultrastructure were evaluated using a transmission electron microscope (HT7700, Hitachi, Japan) by a single electron microscopist.

### 4.9 Serum amylase and lipase measurement

Serum amylase and lipase were measured with assay kits (C016‐1 and A054‐1) from Nanjing Jiancheng Bioengineering Institute (Nanjing, China).

### 4.10 ATP content detection

Pancreatic tissue homogenate was obtained and ATP levels in the pancreatic tissue were assessed by an assay kit (S0026, Beyotime Biotechnology, China).

### 4.11 Enzyme‐linked immunosorbent assay

Seum MFG‐E8 (mouse and human) and LDH levels were measured using corresponding assay kits (SEB286Mu, SEB286Hu, and SEB864Mu) from Cloud‐Clone Corp USCN Life Science, Wuhan, China.

### 4.12 Statistical analysis

Data are analyzed with GraphPad Prism 6.0 Software (San Diego, CA) and expressed as mean ±  SEM (standard error) or SD (standard deviation). The differences between the groups were compared by Student's *t*‐test or one‐way ANOVA and Student Newman Keuls test. Associations between two parameters were analyzed with Spearman's correlation coefficient (ρ). The survival analysis was performed by Kaplan‐Meier method and log‐rank test. A *P*‐value < .05 represented a significant difference.

Methods for TUNEL, DHE (Dihydroethidium) and MitoTracker Staining, Detection of NADH dehydrogenase, SOD, FRAP, and MDA levels, and Western Blot Analysis are provided in the Supporting Information.

## CONFLICT OF INTEREST

The authors declare no conflict of interest.

## AUTHOR CONTRIBUTIONS

Ren Y acquired and analyzed the data, wrote the paper. Liu W, Zhang L, Zhang J, Bi J, Wang T, Wang M, and Du Z participated in data acquirement. Wang Y and Zhang L collected human serum samples. Wu Z and Lv Y interpreted the data. Meng L interpreted the data and revised the paper. Wu R designed and supervised the study and revised the paper. All authors have read and agreed with the final manuscript.

## Supporting information

Supporting Information

Supporting Information

## Data Availability

The data used to support the findings of this study are available from the corresponding author upon request.
