# Supplementary material for: Milk fat globule EGF factor 8 restores mitochondrial function via integrin‐medicated activation of the FAK‐STAT3 signaling pathway in acute pancreatitis
Source: Clin Transl Med. 2021 Jan 24;11(2):e295. doi: 10.1002/ctm2.295 (PMC7828261; doi:10.1002/ctm2.295)
Supplement: Supplementary file 1 — Supporting Information [file CTM2-11-e295-s001.docx]

Supplementary Materials for

**Milk fat globule EGF factor 8 restores mitochondrial function via integrin-medicated activation of the FAK-STAT3 signaling pathway in acute pancreatitis**

Yifan Ren, Wuming Liu, Lin zhang, Jia Zhang, Jianbin Bi, Tao Wang, Mengzhou Wang, Zhaoqing Du, Yawen Wang, Lin zhang, Zheng Wu, Yi Lv, Lingzhong Meng, Rongqian Wu*

*Corresponding author: [rwu001@mail.xjtu.edu.cn](mailto:rwu001@mail.xjtu.edu.cn)

**This PDF file includes:**

Materials and Methods

Supplementary Figure 1

Supplementary Table 1

Supplementary Table 2

**MATERIALS AND METHODS**

**TUNEL, DHE (Dihydroethidium) and MitoTracker Staining:** A TUNEL kit (11684795910, Roche, Switzerland), a DHE (Dihydroethidium, G1045, Servicebio, Wuhan, China) kit and a MitoTracker staining kit (Mitochondrial probe, M7512, Thermo Fisher Scientific, Beijing, China) were used for TUNEL, DHE and MitoTracker staining according to the manufacturers’ instructions.

**NADH dehydrogenase detection:** Mitochondrial complex I activity detection kit (BC0515, Beijing Solarbio Science & Technology Co., Ltd, China) were used for the NADH dehydrogenase detecteion according to the manufacturer’s instructions.

**Detection of SOD, FRAP and MDA levels:** Pancreatic tissue homogenate was obtained and superoxide dismutase (SOD) assay Kit (A001-3, NanJing JianCheng Bioengineering Institute, Nanjing, China), total antioxidant capacity assay kit with FRAP method (Ferric Reducing Antioxidant Power, FRAP) (S0116, Beyotime, Shanghai, China) and malonaldehyde (MDA) assay Kit (A003-1, NanJing JianCheng Bioengineering Institute, Nanjing, China) were used for measuring the levels of MDA, SOD and FRAP in the pancreatic tissue according to the instructions of the kits.

**Western Blot Analysis:** Pancreatic tissues were lysed in cold RIPA (P0013B, Beyotime, Beijing, China). The protein concentration was evaluated with the BCA Protein Assay Kit (P0012S, Beyotime, Beijing, China). After gel electrophoresis, the protein was transferred to PVDF membrane and incubation in blocking solution (3% BSA or 5% skimmed milk) at room temperature. Then the membranes were incubated overnight at 4°C with the primary antibodies (Supplementary Table 2). Primary antibodies were diluted in Primary Antibody Dilution Buffer for Western Blot (P0256, Beyotime, Beijing, China). Membranes were washed and then incubated with specific HRP‐conjugated secondary antibodies (Supplementary Table 2) for 1.5 hours at room temperature. Bands were developed using Digital gel image analysis system (Bio‐Rad, California, USA) and quantitative of protein level were calculated by ImageJ2x software. Information about the antibodies used in this study are listed in Supplementary Table 2.

**Supplementary figure 1. Serum and pancreatic levels of MFG-E8 at different time points after L-arginine-injection in mice.** Arginine-AP was induced by 2 hourly intraperitoneal injections of 4.0 g/kg L-arginine. The animals were sacrificed at 12, 24, 48, 72 and 96 hours after the first injection of L-arginine. (**A**) Serum MFG-E8 levels in AP mice; (**B**) Pancreatic MFG-E8 levels in AP mice. n = 4-7/group, error bars indicate the SEM; ∗ P<0.05 versus Sham group; # P<0.05 versus 24 hours group.

**Supplementary Table 1: Characteristics of patients with acute pancreatitis.**

| **Characteristics** | **Patients**  **N (%) or mean ± SD** | **Volunteers**  **N (%) or mean ± SD** |
| --- | --- | --- |
| Number | 134 | 69 |
| Age (Year) | 47 ± 13.1 | 47.8 ± 14.4 |
| Sex (male/female) | 83/51 | 40/29 |
| Body Mass Index | 22.1 ± 2.3 | 21.8 ± 1.9 |
| Classification |  |  |
| Mild | 80 (59.7%) | N/A |
| Moderately severe | 22 (16.4%) | N/A |
| Severe | 32 (23.9%) | N/A |
| Etiology |  |  |
| Biliary | 57 (42.5%) | N/A |
| Alcoholic | 5 (3.7%) | N/A |
| Hypertriglyceremic | 37 (27.6%) | N/A |
| Others | 35 (26.2%) | N/A |
| Treatments |  |  |
| Conservative therapy | 95 (70.9%) | N/A |
| Percutaneous drainage | 13 (9.7%) | N/A |
| Laparotomy | 26 (19.4%) | N/A |
| APACHE II scores | 5.7 ± 5 | N/A |
| SOFA scores | 2.1 ± 3 | N/A |
| Local complication (Yes/No) | 35/99 | N/A |
| Organ failure (Yes/No) | 19/115 | N/A |
| Serum PCT (ng/ml) | 2.83 ± 6.76 | N/A |
| Serum CRP (mg/L) | 131.5 ± 160 | N/A |
| Serum lipase (U/L) | 1755.6 ± 1837 | N/A |
| Serum amylase (U/L) | 725.4 ± 960.1 | N/A |
| Blood Glucose(mmol/L) | 8.5 ± 7.5 | N/A |
| HbA1c (%) | 5.3 ± 2.1 | N/A |
| Serum K^+^ | 3.9 ± 0.5 | N/A |
| Serum Na^+^ | 138.9 ± 4.0 | N/A |
| PLT | 196.2 ± 73.9 | N/A |
| FIB | 4.6 ± 2.2 | N/A |
| APTT | 37.8 ± 6.9 | N/A |
| WBC(*10^9/L) | 11.6 ± 6.1 | N/A |
| AST | 56.1 ± 81.9 | N/A |
| Serum creatinine(μmol/L) | 85.9 ± 98 | N/A |
| Serum Cr | 71.8 ± 70.6 | N/A |
| Serum BUN (mmol/L) | 7.6 ± 95 | N/A |
| HCT (%) | 37 ± 11.4 | N/A |
| Serum MFG-E8 (ng/ml) | 39.4 ± 18.1 | 67.6 ± 35.5 |

**Supplementary Table 2: Antibodies**

| **Antibody** | **Item No** | **Company and location** |
| --- | --- | --- |
| Anti-PGC1α | ab54481 | Abcam, Cambridge, MA, USA |
| Anti-TFAM | ab131607 | Abcam, Cambridge, MA, USA |
| Mitofusin-2 Rabbit mAb | 9482 | Cell Signaling Technology, Beverly, MA, USA |
| Anti-β-actin | 60008 | proteintech, CN |
| Anti-ATPB antibody | ab14730 | Abcam, Cambridge, MA, USA |
| RIP3 Rabbit mAb | 15828 | Cell Signaling Technology, Beverly, MA, USA |
| Anti-Caspase-3 antibody | ab4051 | Abcam, Cambridge, MA, USA |
| Anti-Cleaved Caspase-3 antibody | ab2302 | Abcam, Cambridge, MA, USA |
| Bax Rabbit mAb | 14796 | Cell Signaling Technology, Beverly, MA, USA |
| Anti-FIS1 antibody | ab71498 | Abcam, Cambridge, MA, USA |
| DRP Rabbit mAb | 8570 | Cell Signaling Technology, Beverly, MA, USA |
| CHOP Mouse mAb | 2895 | Cell Signaling Technology, Beverly, MA, USA |
| Anti-MT-ND3 antibody | Ab192306 | Abcam, Cambridge, MA, USA |
| MFG-E8 antibody | sc-271574 | SANTA CRUZ Biotechnology, Texas, USA |
| Stat3 Rabbit mAb | 12640 | Cell Signaling Technology, Beverly, MA, USA |
| Phospho-Stat3 Antiboty | 9134 | Cell Signaling Technology, Beverly, MA, USA |
| FAK Antiboty | 3285 | Cell Signaling Technology, Beverly, MA, USA |
| Phospho-FAK Antibody | 3283 | Cell Signaling Technology, Beverly, MA, USA |
| Goat anti-Mouse IgG antibody | 31430 | PIONEER Biotechnology, CN |
| Goat anti-Rabbit IgG antibody | 31460 | PIONEER Biotechnology, CN |
