# Supplementary figures and images for: Milk fat globule EGF factor 8 restores mitochondrial function via integrin‐medicated activation of the FAK‐STAT3 signaling pathway in acute pancreatitis
Source: Clin Transl Med. 2021 Jan 24;11(2):e295. doi: 10.1002/ctm2.295 (PMC7828261; doi:10.1002/ctm2.295)

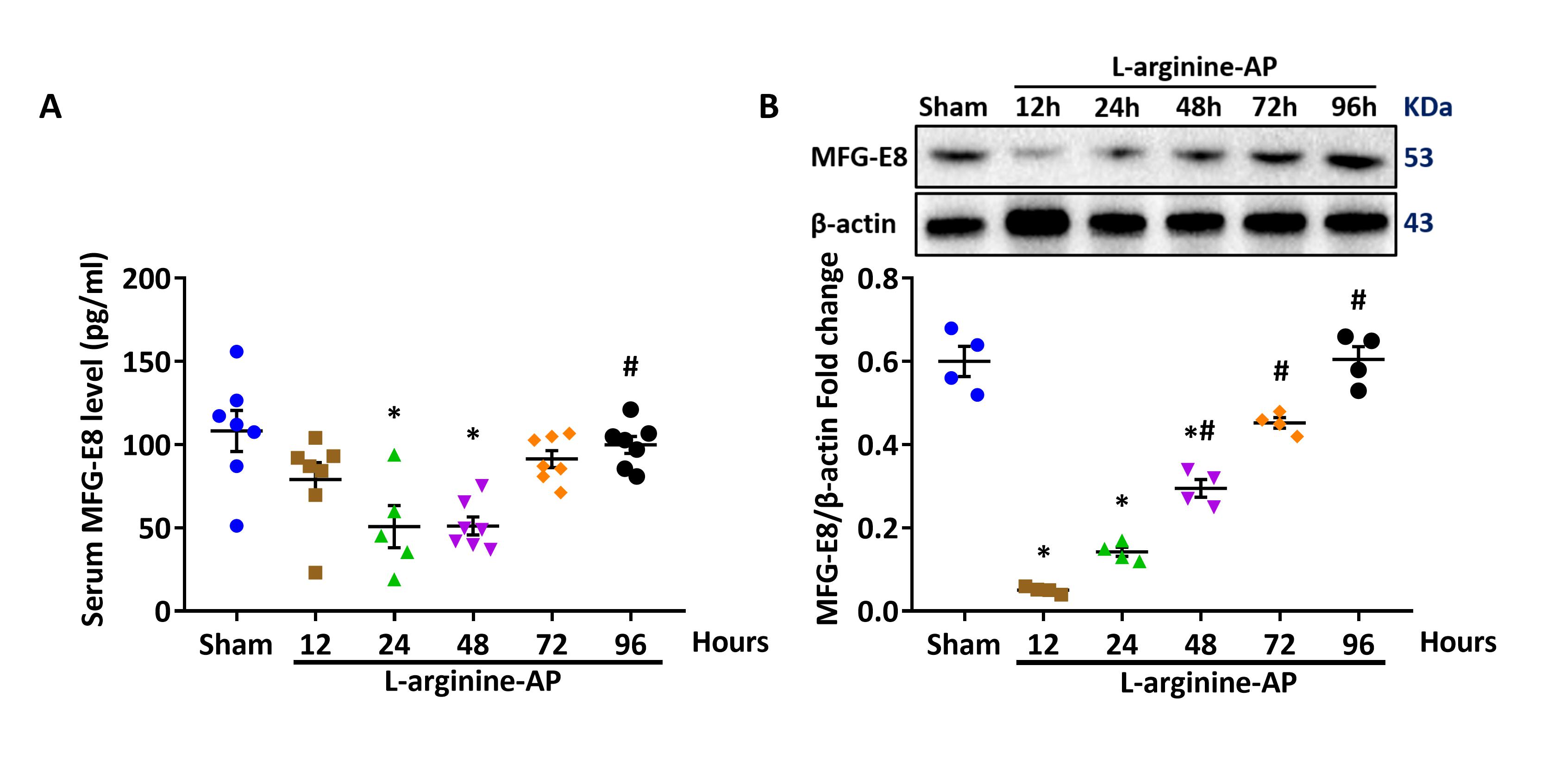

Supplement: Supplementary file 2 — Supporting Information [file CTM2-11-e295-s002.jpg]
